# Biphasic monopolar electrical stimulation induces rapid and directed galvanotaxis in adult subependymal neural precursors

**DOI:** 10.1186/s13287-015-0049-6

**Published:** 2015-04-12

**Authors:** Robart Babona-Pilipos, Alex Pritchard-Oh, Milos R Popovic, Cindi M Morshead

**Affiliations:** Institute of Biomaterials and Biomedical Engineering, University of Toronto, 164 College Street, Room 407, M5S 3G9 Toronto, Ontario Canada; Department of Chemical Engineering, University of Waterloo, Waterloo, ON Canada; Lyndhurst Centre, Toronto Rehabilitation Institute, University Health Network, Toronto, ON Canada; Department of Surgery, University of Toronto, 1 King’s College Circle, Room 1156, M5S 1A8 Toronto, ON Canada; Department of Rehabilitation Science, University of Toronto, 160 College Street, Room 1006, Toronto, ON Canada

## Abstract

**Introduction:**

Following injury such as stroke, adult mammalian subependymal neural precursor cells (NPCs) are induced to proliferate and migrate toward the lesion site where they differentiate into neural cells, albeit with limited efficacy. We are interested in enhancing this migratory ability of NPCs with the long-term goal of promoting neural repair. Herein we build on our previous studies demonstrating that direct current electric fields (DCEFs) promote rapid and cathode-directed migration of undifferentiated adult NPCs (but not differentiated phenotypes) - a phenomenon known as galvanotaxis. While galvanotaxis represents a promising strategy to promote NPC recruitment to lesion sites, stimulation of neural tissue with DCEFs is not a clinically-viable strategy due to the associated accumulation of charge and toxic byproducts. Balanced biphasic waveforms prevent the accumulation of charge and thus are outside of the limitations of DCEFs. In this study, we investigated the effects of balanced biphasic electrical stimulation on the migratory behaviour of undifferentiated subependymal NPCs and their differentiated progeny.

**Methods:**

NPCs were isolated from the subependymal zone of adult mouse brains and cultured in a NPC colony-forming assay to form neurospheres. Neurospheres were plated onto galvanotaxis chambers in conditions that either promoted maintenance in an undifferentiated state or promoted differentiation into mature phenotypes. Chambers containing cells were then time-lapse imaged in the presence of either biphasic monopolar, or biphasic bipolar electrical stimulation, or in the complete absence of electrical stimulation. Single cell migration was subsequently tracked and the cells’ magnitude of velocity, directedness and tortuosity were quantified.

**Results:**

We demonstrate, for the first time, the use of balanced biphasic electric fields to induce galvanotaxis of NPCs. Undifferentiated adult mouse subependymal NPCs exposed to biphasic monopolar stimulation undergo rapid and directed migration toward the cathode. In contrast, both biphasic bipolar stimulation and the lack of electrical stimulation produced non-directed migration of NPCs. Notably, NPCs induced to differentiate into mature phenotypes prior to exposure to electrical stimulation do not migrate in the presence or absence of biphasic stimulation.

**Conclusion:**

We purport that balanced biphasic stimulation represents a clinically-viable technique for mobilizing NPCs that may be integrated into strategies for promoting endogenous neurorepair.

**Electronic supplementary material:**

The online version of this article (doi:10.1186/s13287-015-0049-6) contains supplementary material, which is available to authorized users.

## Introduction

The discovery that neurogenesis persists into adulthood in the mammalian brain has altered our understanding of neuroplasticity and our outlook on repairing the injured brain following injury or disease. Adult neural precursor cells (NPCs) reside in two neurogenic regions in the forebrain: the subependyma lining the lateral ventricles, and the subgranular zone of the hippocampal dentate gyrus [1,2]. Under baseline conditions, subependymal zone (SEZ) NPCs give rise to neuroblasts that migrate along a well-defined pathway known as the rostral migratory stream toward the olfactory bulb, where they differentiate into interneurons. The inherent proliferative, migratory and neurogenic properties of NPCs make them good candidates for contributing to neurorepair following neural insult, such as stroke. Indeed, SEZ-derived NPCs have been shown to contribute to neurogenesis following injury [3]. Interestingly, neural insult alone results in the upregulation of multiple chemical and physical cues that enhance NPC proliferation and induce the redirection of their migration toward the lesion site, as comprehensively reviewed by Kahle and Bix [4]. However, the neuroregenerative impact of endogenous NPC activity is limited. The introduction of exogenous factors can enhance this post-insult response and promote functional recovery [5-7], but long-term safety concerns have limited their clinical applicability. Targeting the recruitment of NPCs to appropriate areas remains a major challenge in neurorepair efforts, and the evolution of novel methods to direct their migration is instrumental to the development of successful neurorepair strategies. NPC migration has most commonly been investigated in the context of chemotaxis. Cytokines such as tumor necrosis factor alpha and stromal cell-derived factor are known regulators of NPC migration [8,9]. Similarly, the expression of growth factors such as vascular endothelial growth factor, epidermal growth factor and basic fibroblast growth factor following neural injury is believed to be involved in the directed migration of NPCs toward damaged areas [5,10,11]. Utilizing such approaches as the basis for the development of therapeutic tools has been hindered by concerns for the long-term safety of such molecules. Given that endogenous electrical fields exist *in vivo* and may serve to mediate endogenous neural precursor migration [12], we are interested in exploring the capacity of exogenously applied electrical fields to direct the migration of NPCs as an alternative to pharmacological approaches.

Endogenous direct current electric fields (DCEFs) play important roles in physiological processes that include development, wound healing, nerve growth and angiogenesis [13-17]. On a cellular level, it has long been known that external application of DCEFs can induce the directed migration of certain cell types toward either the anode or the cathode of the electric field in a process known as galvanotaxis [18-21]. It is interesting that stem cells, including neural stem cells [22,23], human induced pluripotent stem cells and human embryonic stem cells [24], are included within these populations because this raises exciting possibilities of implementing electric fields in regenerative medicine strategies to promote directional migration. We have reported the ability of DCEFs to induce rapid and directed cathodal galvanotaxis of adult SEZ-derived NPCs, but not in differentiated populations [25,26], making the application of electric fields a viable approach to neuroregenerative strategies. However, direct current stimulation may not be suitable for clinical applications. Prolonged exposure to DCEFs results in charge accumulation at the electrode–tissue interface. Such charge build-up produces electrochemical reactions that may cause electrode corrosion, the formation of toxic chemical species and subsequent tissue damage [27,28]. Moreover, excessive charge accumulation at the electrodes could impede the flow of current from the stimulating electrodes [29]. These issues would be ameliorated by the use of balanced biphasic electrical stimulation [28], which is characterized by pulses that consist of both positive and negative phases. Importantly, with balanced biphasic stimulation the electrical charge delivered during one phase would be withdrawn during the opposite phase, resulting in no net charge accumulation and, in principle, the reversal of any electrochemical reactions that could produce toxic byproducts.

Despite these potential benefits over direct current stimulation, successful galvanotaxis of mammalian cells using biphasic stimulation has not yet been reported, and has not been broadly investigated. Chang and colleagues demonstrated that balanced biphasic bipolar (BPBP) pulses promoted expansion and neuronal differentiation of fetal neural precursors [30]. A recent study reported that biphasic stimulation promotes cell survival and anti-apoptotic effects in growth factor-depleted conditions through brain-derived neurotrophic factor and phosphatidylinositol 3′-kinase/Akt signaling [31]. The phosphatidylinositol 3′-kinase/Akt pathway has been implicated as a cellular mechanism by which external application of electric fields is transduced into cellular motility through galvanotaxis [15,32]. Hart and colleagues have demonstrated cathodal keratinocyte galvanotaxis using a combination of direct current and alternating current fields, but alternating fields alone resulted in non-directed, random migration [33]. Herein we report for the first time the use of charge-balanced biphasic electric fields to induce rapid and directed galvanotaxis in clonally derived populations of undifferentiated adult NPCs. Through live cell time-lapse imaging and cell-tracking techniques, we analyze the migratory properties of adult SEZ-derived NPCs and their differentiated progeny. Galvanotaxis was prominent in undifferentiated cells but absent in differentiated populations, reminiscent of our previous work using DCEFs [26]. The present study begins to address a significant concern regarding the utility of galvanotaxis as a therapeutic tool by demonstrating proof-of-principle evidence for the feasibility of inducing directed migration of NPCs through balanced biphasic stimulation. This technology may provide a means for harnessing the therapeutic potential of endogenous NPCs to enhance endogenous neurorepair.

## Methods

### Ethics statement

All animal work was approved by the University of Toronto Animal Care Committee in accordance with institutional guidelines (protocol number 20009955).

### Cell culture

NPCs were isolated and cultured as described previously [34]. Briefly, adult male CD1 mice were sacrificed, and the periventricular regions of the brain were excised and enzymatically dissociated. Cells were plated in serum-free media (SFM) (Dulbecco’s modified Eagle’s medium:F12, 3:1; Invitrogen, Burlington, ON, Canada) supplemented with epidermal growth factor (20 ng/ml; Sigma-Aldrich, Oakville, ON, Canada), basic fibroblast growth factor (10 ng/ml; Sigma-Aldrich) and heparin (2 μg/ml; Sigma-Aldrich) – herein referred to as SFM + EFH – at 10 cells/μl in T25 culture flasks (BD Biosciences, Mississauga, ON, Canada) [35,36]. After 7 days in culture primary neurospheres (passage 0) consisting of nestin-expressing NPCs were collected and plated for galvanotaxis experiments or mechanically dissociated and replated in identical neurosphere forming conditions to form secondary neurospheres (passage 1). Neurospheres were passaged and plated in mitogenic conditions every 7 days. Neurospheres up to passage 4 were utilized for experiments.

### Galvanotaxis chamber construction

Galvanotaxis chambers were constructed as described previously [25]. Briefly, square no. 1 glass cover slides (22 mm × 22 mm × 0.17 mm; VWR, Mississauga, ON, Canada) were acid-washed in 6 N HCl overnight, then sealed to the base of 60 mm × 15 mm Petri dishes (VWR) using silicone vacuum grease (VWR). Other square no. 1 glass slides were then cut into rectangular strips (22 mm × 4 mm × 0.17 mm), washed with 70% ethanol followed by autoclaved water, and sealed with grease to opposite edges of the square slide in the Petri dish. The resulting central trough measured 22 mm × 12 mm × 0.5 mm (length × width × height, accounting for the thickness of the grease). Chambers were UV sterilized, and the central troughs coated with poly-l-lysine (100 μg/ml; Sigma, Canada) for 2 hours at room temperature, followed by 4% (v/v) Matrigel (BD Biosciences, Canada) in SFM for 1 hour at 37°C. Next, 350 μl of either SFM + EFH or SFM supplemented with 1% fetal bovine serum (FBS; Life Technologies, Burlington, ON, Canada) – herein referred to as SFM + FBS – were added to the central troughs of the chambers with five or six neurospheres that were 80 to 100 μm in diameter. Chambers were then incubated at 37°C, 5% carbon dioxide and 100% humidity. To investigate the galvanotaxis of undifferentiated NPCs, neurospheres were plated onto the chambers for 17 to 20 hours in SFM + EFH to allow the neurosphere cells to adhere to the Matrigel matrix while remaining undifferentiated. In contrast, to investigate the galvanotaxis of differentiated NPCs, neurospheres were plated onto the chambers for 72 to 96 hours in SFM + FBS to allow neurosphere cells to adhere and differentiate into mature neural phenotypes.

### Galvanotaxis assay

Two 15 cm long pieces of PVC tubing (2.38 mm i.d., 3.97 mm o.d.; Fisher Scientific, Mississauga, ON, Canada) were filled with 1.5% (w/v) agarose gel. Ag/AgCl electrodes were formed by coiling and immersing two 10 cm pieces of silver wire (Alfa Aesar, Ward Hill, MA, USA) in bleach for 20 minutes. A square no. 1 glass cover slide was sealed with grease to the top of the rectangular strips on either side of the trough, creating a central chamber. Strips of grease were used to separate pools of media on either side of the central chamber. The galvanotaxis chamber was transferred onto the stage of a temperature-controlled, carbon dioxide-controlled and humidity-controlled Zeiss Observer Z1 microscope (Zeiss, Oberkochen, Germany) for time-lapse imaging. Two 60 mm × 15 mm Petri dishes were placed on the stage – one on either side of the galvanotaxis chamber – and filled with 7.5 ml SFM. The Ag/AgCl electrodes were placed into the peripheral Petri dishes, and all three dishes were bridged with the agarose gel tubes to establish electrical continuity. The Compex Motion electrical stimulator (developed at the Rehabilitation Engineering Laboratory, University of Toronto, ON, Canada; discussed further below) was connected to the Ag/AgCl electrodes for biphasic pulse application. Cells were electrically stimulated for 2.5 to 6 hours during which time-lapse imaging was performed via Zeiss Axiovision software to record cell migration at a capture rate of one image per minute.

The voltage across the galvanotaxis chamber was measured using a LabQuest 2 (Vernier, Beaverton, OR, USA) data acquisition unit equipped with a Vernier differential voltage sensor. This apparatus was also used to obtain traces of the biphasic stimulator’s output waveforms. Output waveforms of 400 Hz frequency were captured for 0.5 seconds at a sampling rate of 20,000 samples per second. The peak cathodal potential difference across the galvanotaxis chamber was measured for each captured cycle to obtain the mean peak cathodal potential over the 0.5 second period. This value was divided by the length of the chamber (22 mm) to obtain the mean peak electrical field strength. The waveform traces were analyzed for the area under the curves using Prism software (GraphPad Software, La Jolla, CA, USA).

### Kinematics of cell migration

Zeiss Axiovision software was used to track cell migration. At least 45 cells from three independent experiments were tracked for each experimental group. Cells were selected for tracking if they were at least one cell body away from the nearest cell at the beginning of the experiment (that is, time = 0). Cell centroid positions were used for tracking cell migration. The magnitude of velocity (|velocity|), directedness and tortuosity of migration were analyzed. The |velocity| of each cell was obtained by dividing the total displacement of a cell from its initial position (at time 0) to its final position by the total time taken to arrive at this latter position. The directedness of a cell was obtained by finding the cosine of the angle between the positive *x* axis (the direction of the cathodal electric field vector) and the straight-line displacement between the cell’s original position and its final position. The tortuosity was obtained by dividing the total path length traversed by the cell by the straight-line displacement between the cell’s initial position at time 0 and its final position.

### Immunocytochemistry

Cells were fixed for 10 minutes at room temperature with 4% paraformaldehyde, and then triple washed with phosphate-buffered saline for 5 minutes each time. Cells were permeabilized for 20 minutes at room temperature with 0.3% Triton X-100, and then blocked with 10% normal goat serum (Jackson Immunoresearch Laboratories, Burlington, ON, Canada) in phosphate-buffered saline for 1 hour at room temperature. Samples were processed with primary antibodies for NPCs (mouse monoclonal anti-Nestin, 1:400; Millipore, Etobicoke, ON, Canada), neurons (rabbit polyclonal anti-class III β-tubulin; Covance, Princeton, NJ, USA) and astrocytes (rabbit polyclonal anti-glial fibrillary acidic protein, 1:500; Sigma) overnight at 4°C. Samples were triple washed with phosphate-buffered saline for 5 minutes, and incubated with appropriate secondary antibodies for 1 hour at 37°C. Secondary antibodies used were Alexafluor 568 (goat anti-mouse, 1:400; Life Technologies), Alexafluor 488 (goat-anti-rabbit, 1:400; Life Technologies) and Alexafluor 488 (goat anti-mouse, 1:300; Life Technologies). Mounting medium containing 4′,6-diamidino-2-phenylindole (Vector Laboratories, Burlington, ON, Canada) was used for nuclear staining.

### Measurements of media conductivity

Culture media conductivity measurements were recorded with a conductivity meter (CDM80; Radiometer, Copenhagen, Denmark) according to the manufacturer’s protocol. The media were heated to 37°C prior to conductivity measurement. The mean conductivity was determined from three measurements taken on separate days from different batches of media.

### Statistical analysis

Values are presented as group means ± standard error of the mean. Differences between group means were determined using one-way analysis of variance, followed by Bonferroni *post hoc* analyses. Statistical significance was set at *P* <0.05.

## Results

All investigations in this study utilized the Compex Motion electrical stimulator, developed by the Rehabilitation Engineering Laboratory at the University of Toronto, in collaboration with Compex SA (developer of electrostimulation products, Mississauga, ON, Canada) and the Automatic Control Laboratory from the Swiss Federal Institute of Technology, Zurïch, Switzerland. The Compex Motion is a portable electrical stimulator used for functional electrical stimulation therapy, which has been shown in several studies and clinical trials to improve motor function in individuals with spinal cord injury [37-43]. The Compex Motion has four current regulated output channels, each of which are independently controlled. The device’s output waveforms are programmed and stored on chip cards, which can be easily exchanged to modify the stimulator’s output function.

**Figure 1:**
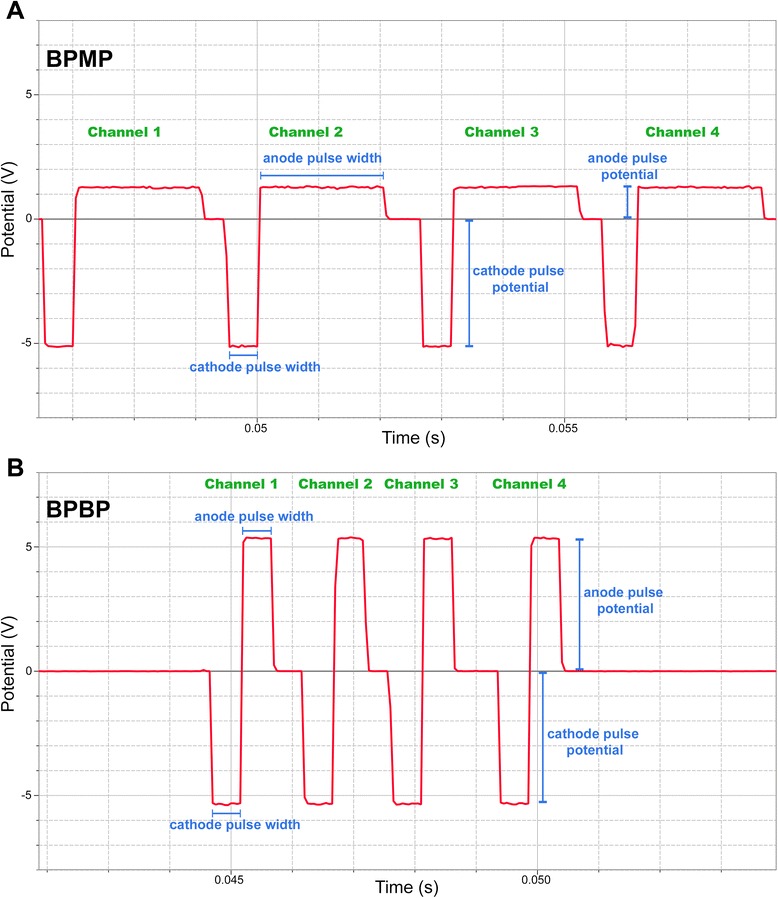
Oscilloscope traces of representative biphasic monopolar and biphasic bipolar pulses. **(A)** A typical biphasic monopolar (BPMP) pulse train with a negative cathodal phase that is four times the amplitude and one-quarter the duration of the balancing positive anodal phase. **(B)** A typical biphasic bipolar (BPBP) pulse train with a negative cathodal phase that is equal in amplitude and duration but opposite in direction to the opposing anodal phase. This demonstrates the relationship between pulse amplitudes and durations between positive (anode) and negative (cathode) phases.

The stimulator’s output functions can be classified under three operational modes: biphasic monopolar (BPMP), the negative phase of each pulse is balanced by a positive phase that is one-quarter the amplitude and four times the duration (Figure 1A); BPBP, the negative phase of each pulse is balanced by a positive phase that is equal in amplitude and duration but opposite in polarity (Figure 1B); and monophasic monopolar, each pulse consists of a single negative phase, lacking a positive phase, and therefore this mode is not charge balanced. The monophasic monopolar mode delivers unidirectional current, which is comparable with DCEF stimulation, and therefore this mode was not investigated in the present study.

For cells to undergo directionally biased galvanotaxis in the presence of balanced biphasic electric fields, the electrical pulses must elicit a galvanotactic response in one phase, but not in the opposite balancing phase. We proceeded by assuming that there exists a threshold level of current below which galvanotaxis would not be induced, consistent with our observations in earlier unpublished work. Our previous work demonstrated rapid and directed cathodal migration in a DCEF [25,26]. Accordingly, we designed a BPMP waveform such that the negative (cathodal) phase of the pulse would induce galvanotaxis, but the positive (anodal) balancing phase of the pulse (which is one-quarter the amplitude and four times the duration of the cathodal phase) would possess a current amplitude below this threshold, and would therefore not create a galvanotactic effect in the opposing direction. A cathodal amplitude of 4 mA in BPMP mode would result in an amplitude of 1 mA during the opposing anodal phase. Since our previous NPC galvanotaxis work with DCEFs utilized current amplitudes of 1 to 2 mA [25,26], we reasoned that an amplitude <1 mA during the anodal phase would be necessary to ensure that opposing anode galvanotaxis would not occur. Based on the above criteria and through a trial and error process (Additional file 1), we utilized the following parameters to create the BPMP and BPBP waveforms used throughout the study. BPMP waveforms were characterized by a cathodal current amplitude of 3 mA and pulse width of 500 microseconds, followed by an anodal current amplitude of 0.75 mA and pulse width of 2,000 microseconds, resulting in a total pulse duration of 2,500 microseconds and duty cycle of 20%. In BPBP mode, a cathodal phase with 500 microseconds pulse width and 3 mA amplitude is followed by a balancing anodal phase of 500 microseconds pulse width and 3 mA amplitude, for a total pulse duration of 1,000 microseconds and a duty cycle of 50%. The maximum frequency of each output channel is 100 Hz. However, the outputs of the four channels are staggered (Figure 1), and therefore the channels were connected in parallel to achieve maximum stimulation frequency, resulting in both BPMP and BPBP waveforms operating at a frequency of 400 Hz. This allowed the cells to receive maximum exposure to the cathodal current without the overlapping of channel outputs.

The theoretical electric field experienced by the cells within the chamber during the cathodal phase can be derived by Ohm’s law for conductive media: E = J/σ, where E is the strength of the electric field, J is the current density (total current through the chamber divided by the chamber’s cross-sectional area) and σ is the conductivity of the culture medium [44]. A current of 3 mA driven through chambers with cross-sectional area of ~7 mm^2^ (14 mm × ~0.5 mm) results in a current density of ~0.428 mA/mm^2^. The conductivity of the culture medium at 37°C was measured as 14.86 ± 0.60 mS/cm, or equivalently the resistivity was 673.38 ± 27.14 Ωmm. By substituting the values of current density and mean conductivity into the above equation, a calculated value of ~288 mV/mm is obtained for the strength of the electric field. This represents the field strength during the cathodal phase of the BPMP waveform, as well as during both phases of the BPBP waveform, and is close to the 250 mV/mm field strength utilized in previous work with DCEFs by our group and others [25,26].

We measured the mean peak strength of the cathodal electrical field across the galvanotaxis chamber using a LabQuest 2 data acquisition unit equipped with a differential voltage sensor. The Compex stimulator’s output waveforms were captured at a sampling rate of 20,000 samples per second for a duration of 0.5 seconds. At a stimulation frequency of 400 Hz, this results in the tracing of approximately 200 cycles. The mean peak cathodal electric field strength during BPMP and BPBP stimulation was measured as 234 mV/mm and 244 mV/mm, respectively (Table 1). We also measured the area under the curve of the BPMP and BPBP waveform traces (with cathodal voltage measured as negative area and anodal voltage measured as positive area) to ensure that the stimulator’s output waveforms are indeed charge balanced (Table 1). A perfectly charge-balanced system would produce a net area under the curve of zero.

**Table 1:** **Analysis of the area under the curve and the mean peak cathodal electrical field strength for BPMP and BPBP pulses**

**Stimulation mode**	**Cycles analyzed**	**Total positive AUC (V•second)**	**Total negative AUC (V•second)**	**Magnitude of net area**	**Peak cathodal shield strength (mV/mm)**
BPMP	160	0.4164	0.4130	0.0034	234
BPBP	201	0.5237	0.5270	0.0033	244

AUC, area under the curve; BPBP, biphasic bipolar; BPMP, biphasic monopolar.

### Biphasic monopolar pulses induce rapid and directed galvanotaxis in undifferentiated NPCs

**Figure 2:**
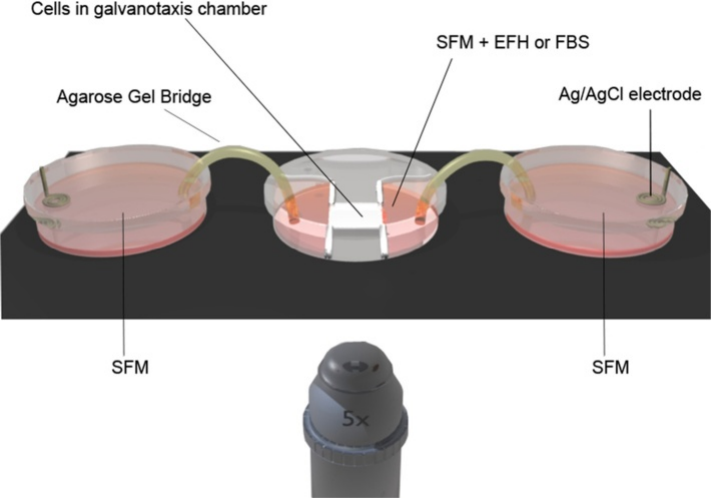
Illustration of experimental setup for galvanotaxis assay. Neural precursor cells plated in a galvanotaxis chamber are placed in the center of the stage of a live-cell imaging system. On either side of the chamber is a Petri dish containing serum-free media (SFM) and housing the Ag/AgCl electrode. The adjacent Petri dishes and the galvanotaxis chamber are bridged via agarose gel electrodes. The Compex stimulator is connected to the Ag/AgCl electrodes to provide biphasic monopolar or biphasic bipolar stimulation. Modified with permission from Babona-Pilipos and colleagues [25]. EFH, epidermal growth factor, basic fibroblast growth factor and heparin; FBS, fetal bovine serum.

We asked whether BPMP pulses could elicit a galvanotactic response in undifferentiated NPCs. We plated neurospheres (passages 0 to 4) onto galvanotaxis chambers for 17 to 20 hours in the presence of growth factors (SFM + EFH) to maintain the NPCs in an undifferentiated state. During this period, neurospheres adhered to the Matrigel-coated base of the chambers and individual NPCs moved radially away from the plated neurosphere. The migration of undifferentiated NPCs was then analyzed using time-lapse imaging for a period of 2.5 to 6 hours in the presence of BPMP or BPBP stimulation, as well as without any applied electric field. Figure 2 illustrates the experimental setup of the apparatus.

**Figure 3:**
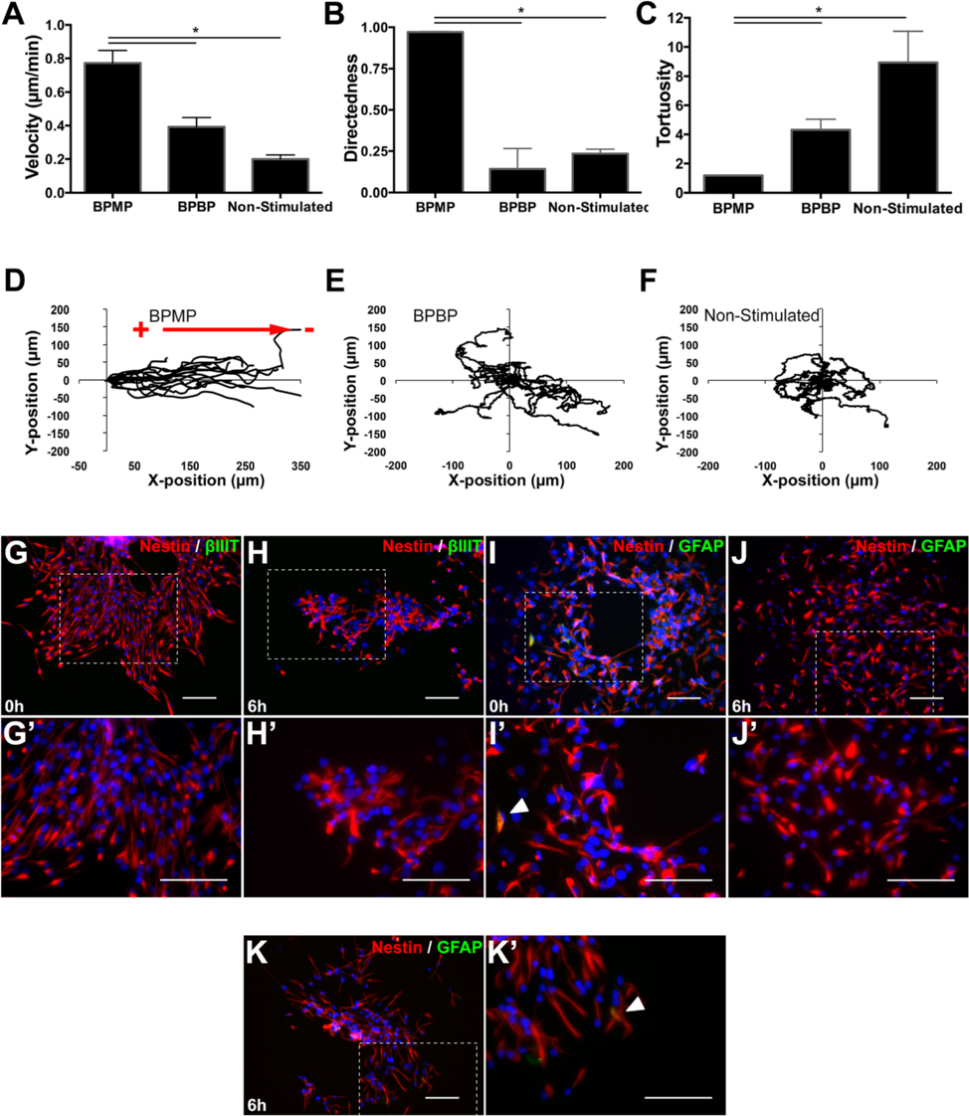
Balanced biphasic monopolar stimulation induces rapid and cathode-directed galvanotaxis in undifferentiated neural precursor cells. **(A, B, C)** Neural precursor cells (NPCs) exposed to biphasic monopolar (BPMP) stimulation (*n* = 3) have a greater magnitude of velocity (|velocity|) **(A)** and directedness **(B)** and a lower tortuosity **(C)** of migration compared with nonstimulated NPCs (*n* = 3), and a greater |velocity| **(A)** and directedness **(B)** compared with biphasic bipolar (BPBP)-stimulated groups (*n* = 3). **(D, E, F)** Individual cell migration tracks localized to a common origin show that undifferentiated NPCs migrate toward the cathode terminal in the presence of BPMP pulses **(D)**, but undergo nondirected migration in the presence of BPBP stimulation **(E)** or in the absence of stimulation **(F)**. **(G, H, I, J, K)** NPCs maintained in the presence of epidermal growth factor, basic fibroblast growth factor and heparin express nestin, but not β-III tubulin **(G, H)**, or glial fibrillary acidic protein (GFAP) **(I, J)**, prior to **(G, I)** and following **(H, J)** 6 hours of time-lapse imaging when exposed to biphasic electrical stimulation, or when not stimulated **(K)**. **(G′, H′, I′, J′, K′)** Higher magnification images of the regions within the dashed boxes in **(G)** to **(K)**. Scale bars = 100 μm. Data presented as mean ± standard error of the mean. *P <0.05.

In the absence of electrical stimulation, undifferentiated NPCs migrated at an overall mean |velocity| of 0.20 ± 0.02 μm/minute (Figure 3A). Their migration was nondirected, with an overall mean directedness of 0.23 ± 0.03 and a mean tortuosity of 8.92 ± 2.15 (Figure 3B,C,F; Additional file 2). Cells exposed to BPBP stimulation exhibited similarly nondirected and tortuous migratory behavior (Figure 3B,C,E) but with a greater overall mean |velocity| of 0.39 ± 0.06 μm/minute (Figure 3A; Additional file 3). Interestingly, this suggests that although BPBP stimulation has no impact on the cells’ direction of migration, it still increases their rate of migration.

Strikingly, when undifferentiated NPCs were exposed to BPMP stimulation they underwent rapid galvanotaxis at a rate of 0.78 ± 0.07 μm/minute (a significant twofold and threefold increase as compared with BPBP-stimulated and nonstimulated groups, respectively, *P* <0.05) (Figure 3A). The migration of cells in the presence of BPMP stimulation was highly cathodally directed, with mean overall directedness of 0.98 ± 0.01 (>8-fold and >4-fold increase compared with BPBP-stimulated and nonstimulated groups, *P* < 0.05) and mean overall tortuosity of 1.18 ± 0.03 (>7-fold decrease compared with nonstimulated groups, *P* < 0.05) (Figure 3B,C,D; Additional file 4). This analysis was also performed in 30-minute increments throughout the time lapse (Additional file 5). Notably, undifferentiated NPCs exposed to BPMP stimulation migrated at approximately 75% of |velocity| and 100% of directedness of NPCs exposed to DCEFs in our previous work [26], even though BPMP-stimulated cells had only 20% of the exposure to cathodal current as compared with DCEF-stimulated cells. Out of 674 cells analyzed across three independent experiments, 95.9 ± 0.01% of undifferentiated NPCs underwent cathodal galvanotaxis in the presence of BPMP stimulation. Hence, undifferentiated NPCs exposed to BPMP pulses undergo rapid and directed galvanotaxis toward the cathode, but their migration is slower and nondirected when either exposed to BPBP pulses or in the absence of electrical stimulation.

Prestimulation and poststimulation immunocytochemical analysis verified that the cells remained nestin^+^ (a marker of undifferentiated NPCs) and did not express neuronal (β-III tubulin) or glial (glial fibrillary acidic protein) markers, when maintained in the presence of growth factors, regardless of whether or not they were exposed to biphasic stimulation (Figure 3G,H,I,J,K). A small subset of cells coexpressed glial fibrillary acidic protein and nestin (arrowhead in Figure 3I′,K′), and these may represent *bona fide* type B neural stem cells [45].

### Differentiated neural cells do not undergo galvanotaxis

**Figure 4:**
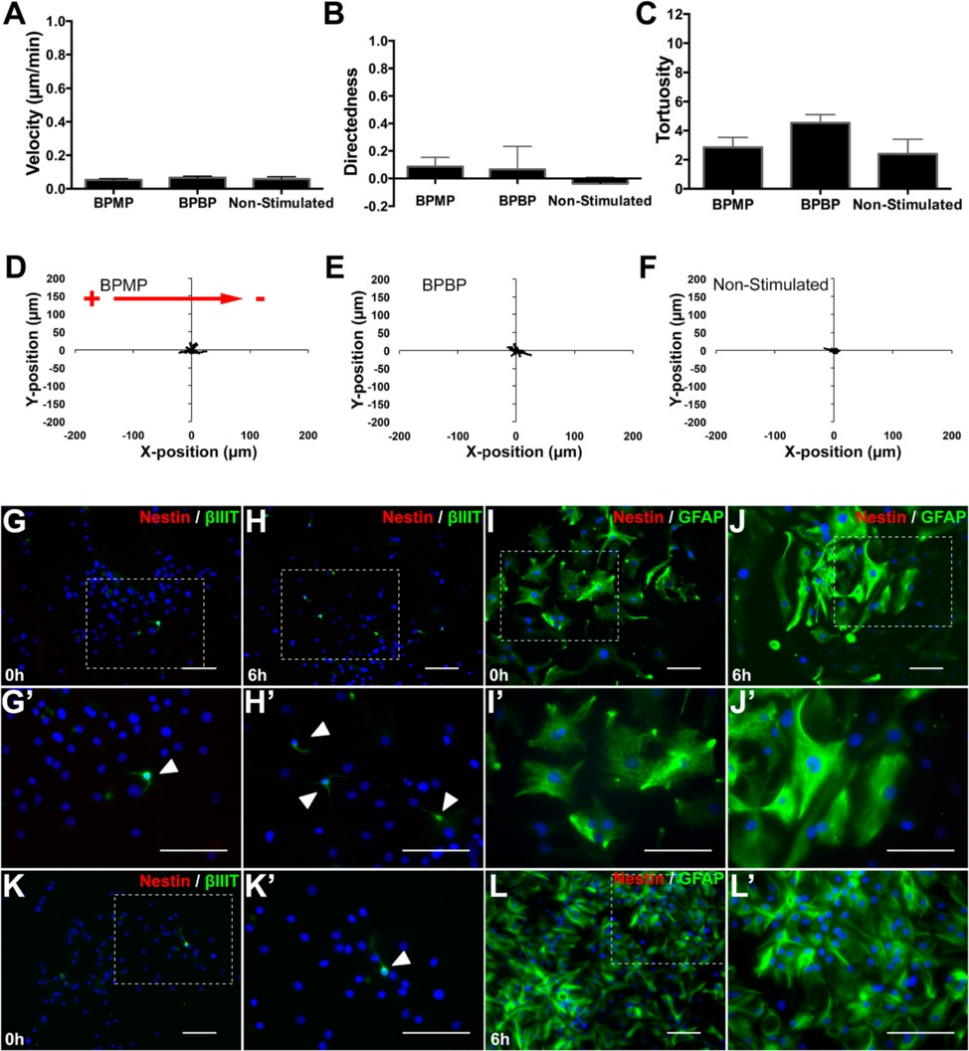
Balanced biphasic stimulation does not elicit galvanotaxis in neural precursor cells induced to differentiate. **(A, B, C)** Differentiated cells exhibit similar magnitude of velocity (|velocity|) **(A)**, directedness **(B)** and tortuosity **(C)** of migration when exposed to biphasic monopolar (BPMP) stimulation (*n* = 3), biphasic bipolar (BPBP) stimulation (*n* = 3) or no stimulation (*n* = 3). **(D, E, F)** Individual cell migration tracks localized to a common origin show that differentiated cells undergo little migration over 6 hours of time-lapse imaging in the presence of BPMP stimulation **(D)**, BPBP stimulation **(E)** or in the absence of stimulation **(F)**. **(G, H, I, J, K, L)** Differentiated cells express β-III tubulin (**G**, **H**; arrowheads) or glial fibrillary acidic protein **(GFAP)** **(I, J)** prior to **(G, I)** and following **(H, J)** 6 hours of time-lapse imaging, when exposed to biphasic electrical stimulation, or not stimulated **(K, L)**. **(G′, H′, J′, K′, L′)** Higher magnification images of the regions within the dashed boxes in **(G to L)** *.* Scale bars = 200 μm. Data presented as mean **±** standard error of the mean. *P <0.05.

We previously demonstrated that when NPCs are induced to differentiate into neural phenotypes, they lose their ability to undergo galvanotactic migration in the presence of DCEFs [26]. We next asked whether differentiated cells would undergo galvanotaxis when exposed to BPMP or BPBP pulses. Neurospheres (passages 0 to 4) were plated onto Matrigel-coated galvanotaxis chambers in the presence of 1% FBS for 72 to 96 hours to induce cell differentiation. Differentiated cells were maintained in 1% FBS conditions and time-lapse imaged for 2.5 to 6 hours while either stimulated with BPMP or BPBP pulses, or not stimulated. Differentiated cells did not undergo galvanotaxis either in the presence or the absence of biphasic stimulation (both BPMP and BPBP modes), consistent with our previous observations with DCEFs. The |velocity| (BPBP: 0.07 ± 0.01 μm/minute, BPMP: 0.06 ± 0.01 μm/minute), directedness (BPBP: 0.07 ± 0.17, BPMP: 0.09 ± 0.07) and tortuosity (BPBP: 4.53 ± 0.58, BPMP: 2.85 ± 0.68) of migration did not significantly differ from that of nonstimulated differentiated cells (|velocity|: 0.06 ± 0.01 μm/minute, directedness: −0.04 ± 0.04, tortuosity: 2.39 ± 1.01) (Figure 4A,B,C,D,E,F; Additional file 6). Similar to undifferentiated cells, this analysis was repeated throughout the entire time lapse in 30-minute increments (Additional file 7). Immunocytochemical analysis verified that the cells had differentiated into mature phenotypes prior to time-lapse imaging, and their mature phenotypes were maintained after 6 hours of imaging in the presence or absence of biphasic stimulation (Figure 4G,H,I,J,K,L). We conclude that biphasic stimulation does not induce galvanotaxis in differentiated neural cells.

## Discussion

Effective endogenous neural repair strategies require efficient and controlled methods of mobilizing resident NPCs from their niche toward injured regions. The concept of using electric fields to promote neuroregeneration has been proposed as a viable clinical approach [14]. To date, studies in the literature that investigate the effects of electric fields on cells utilize DCEFs, and hence their clinical applicability is limited due to the damaging effects of DCEFs on neural tissue [28,46]. Electrochemical reactions that occur at the electrode–tissue interface produce byproducts that elicit cytotoxic effects [47]. Balanced biphasic stimulation, in contrast, is not encumbered with this limitation and thus represents a more clinically relevant approach to pursuing galvanotaxis-based therapies. Despite this, however, there have been no prior reports of successful galvanotaxis in any mammalian cell populations using balanced biphasic electric fields.

Here we report for the first time that the delivery of balanced BPMP pulses induces rapid and directed cathodal galvanotaxis (that is, whole cell body translocation in the presence of electric fields) in undifferentiated adult SEZ-derived NPCs. To our knowledge, this is the first report of biphasic electric fields inducing galvanotaxis in a mammalian cell population. We also demonstrate that galvanotaxis does not occur in NPCs exposed to BPBP pulses, in which the electrical pulses are completely symmetrical. We propose that the asymmetrical nature of BPMP pulses allows the cathodal phase of the pulses to elicit an effect that is not observed during the anodal phase, thus producing a cellular response that is biased to one phase of the electric field. Conversely, symmetrical BPBP pulses would presumably elicit identical cellular responses during each phase but in opposing directions, yielding no net bias in migratory behavior. Moreover, we demonstrate that both BPBP and BPMP stimulation fail to produce a galvanotactic effect in NPCs that have been induced to differentiate into mature phenotypes, implying that balanced biphasic stimulation can selectively target undifferentiated NPC populations. These findings are consistent with our previous work demonstrating that clonally derived populations of adult NPCs undergo rapid cathodal galvanotaxis when exposed to DCEFs of strength 250 mV/mm [26].

Remarkably, the BPMP-stimulated cells in the present study migrate at 100% of the directedness and 71% of the |velocity| of cells that are exposed to DCEF stimulation, considering that BPMP-stimulated cells had only 20% of the exposure to cathodal current as compared with DCEF-stimulated cells. There are infinite combinations of amplitude, pulse width and frequency settings available for designing BPMP pulses, and it is conceivable that NPC migration velocity might be enhanced further by determining the optimal pulse parameter values.

Biphasic electrical stimulation is a well-established clinical tool that has beneficial applications in neuromodulation, cardiac pacing and deep brain stimulation [48,49]. We propose that the clinical utility of biphasic stimulation may expand to include the directed recruitment and migration of endogenous or transplanted NPCs, and possibly other stem cell populations. In comparison with other cell recruitment strategies that have been explored such as chemotactic migration [50,51], electrical stimulation offers the benefit of easy control over the strength, duration and direction of application. One must consider that the cell behavior observed *in vitro* may not accurately reflect their *in vivo* migratory capacity, which would be influenced by cell–cell and cell–extracellular matrix interactions, cell surface receptor expression, and gradients in various signaling molecules [20]. Our future work will investigate the ability of BPMP stimulation to induce NPC migration *in vivo*, and whether electric field-enhanced recruitment of NPCs toward damage sites in an injury model impacts tissue repair and functional recovery.

The discovery of neural stem cells in the adult brain has led to extensive investigation of their potential for promoting endogenous repair following neural injury or disease. Although neural insult alone is sufficient to expand resident NPC populations, and although this process can be augmented with exogenous factors, only a subpopulation of the expanded pool migrates to lesion sites in response to insult [52]. We propose that endogenous neurorepair processes may be enhanced by recruiting greater numbers of NPCs toward insult regions, and our data suggest that balanced biphasic electrical stimulation may represent a clinically relevant means to accomplish this by selectively targeting and controlling the directed migration of undifferentiated NPCs.

## Conclusion

The principal finding of this study is that balanced BPMP electrical waveforms can induce the galvanotaxis of undifferentiated NPCs, but not differentiated phenotypes. This directed migratory behavior is absent in undifferentiated neural precursors both when stimulated with BPBP waveforms and when not electrically stimulated. The characteristic charge-balanced nature of BPMP waveforms makes them a clinically relevant means of electrically stimulating neural tissue without the toxic accumulation of charge that is associated with direct current stimulation. Further work is required to evaluate the effectiveness of BPMP stimulation in evoking NPC galvanotaxis *in vivo.*

## Additional files

Additional file 1:
**is a table summarizing BPMP pulse parameters that failed to induce NPC galvanotaxis.** Biphasic pulses consisting of these parameters failed to induce NPC galvanotaxis. Additional file 2:
**is a video showing that nonstimulated NPCs undergo nondirection migration.** NPCs were plated into galvanotaxis chambers for 17 to 20 hours in the presence of EFH, and their migration was subsequently imaged in the absence of applied electrical stimulation. Under these conditions, undifferentiated NPCs migrate in a nondirected manner. 1 second of video = 15 minutes of real time. Additional file 3:
**is a video showing that NPCs stimulated with BPBP pulses undergo nondirection migration.** NPCs were plated into galvanotaxis chambers for 17 to 20 hours in the presence of EFH, and their migration was subsequently imaged in the presence of BPBP pulses. Under these conditions, undifferentiated NPCs migrate in a nondirected manner, similar to NPCs in the absence of electrical stimulation. 1 second of video = 15 minutes of real time. Additional file 4:
**is a video showing that NPCs stimulated with BPMP pulses undergo rapid and cathode-directed galvanotaxis.** NPCs were plated into galvanotaxis chambers for 17 to 20 hours in the presence of EFH, and their migration was subsequently imaged in the presence of BPMP pulses. Under these conditions, undifferentiated NPCs migrate in a rapid and cathode-directed manner. 1 second of video = 15 minutes of real time. Additional file 5:
**is a figure showing that undifferentiated NPCs undergo rapid and cathode-directed galvanotaxis in the presence of BPMP stimulation.** (A, B, C) The |velocity| (A), directedness (B) and tortuosity (C) of migration of undifferentiated NPCs are plotted in 30-minute intervals over the course of time-lapse imaging. The average |velocity|, directedness, and tortuosity at each time point t_x_, where x is in increments of 30 minutes, were calculated based on t = 0 as the initial time point. Tracking was performed for up to 210 minutes – the maximum amount of time that was common between all undifferentiated NPC groups. *n* = 3 for each group, **P* <0.05. Additional file 6:
**is a video showing that differentiated NPCs stimulated with biphasic pulses do not undergo migration.** NPCs were plated into galvanotaxis chambers for 72 to 96 hours in the presence of 1% FBS and allowed to differentiated. Their migration was subsequently imaged in the presence of BPMP pulses. Under these conditions, differentiated NPCs undergo negligible migration. 1 second of video = 15 minutes of real time. Additional file 7:
**is a figure showing that differentiated cells undergo negligible migration in the presence and absence of balanced biphasic stimulation.** (A, B, C) The |velocity| (A), directedness (B), and tortuosity (C) of migration of differentiated cells are plotted in 30-minute intervals over the course of time-lapse imaging The average |velocity|, directedness, and tortuosity at each time point t_x_, where x is in increments of 30 minutes, were calculated based on t = 0 as the initial time point. Tracking was performed for up to 150 minutes – the maximum amount of time that was common between all differentiated cell groups. *n* = 3 for each group, **P* <0.05.

## Abbreviations

BPBP: biphasic bipolar
BPMP: biphasic monopolar
DCEF: direct current electric field
EFH: epidermal growth factor, basic fibroblast growth factor and heparin
FBS: fetal bovine serum
NPC: neural precursor cell
SEZ: subependymal zone
SFM: serum-free media
|velocity|: magnitude of velocity
