# Supplementary figures and images for: Biphasic monopolar electrical stimulation induces rapid and directed galvanotaxis in adult subependymal neural precursors
Source: Stem Cell Res Ther. 2015 Apr 12;6(1):67. doi: 10.1186/s13287-015-0049-6 (PMC4413998; doi:10.1186/s13287-015-0049-6)

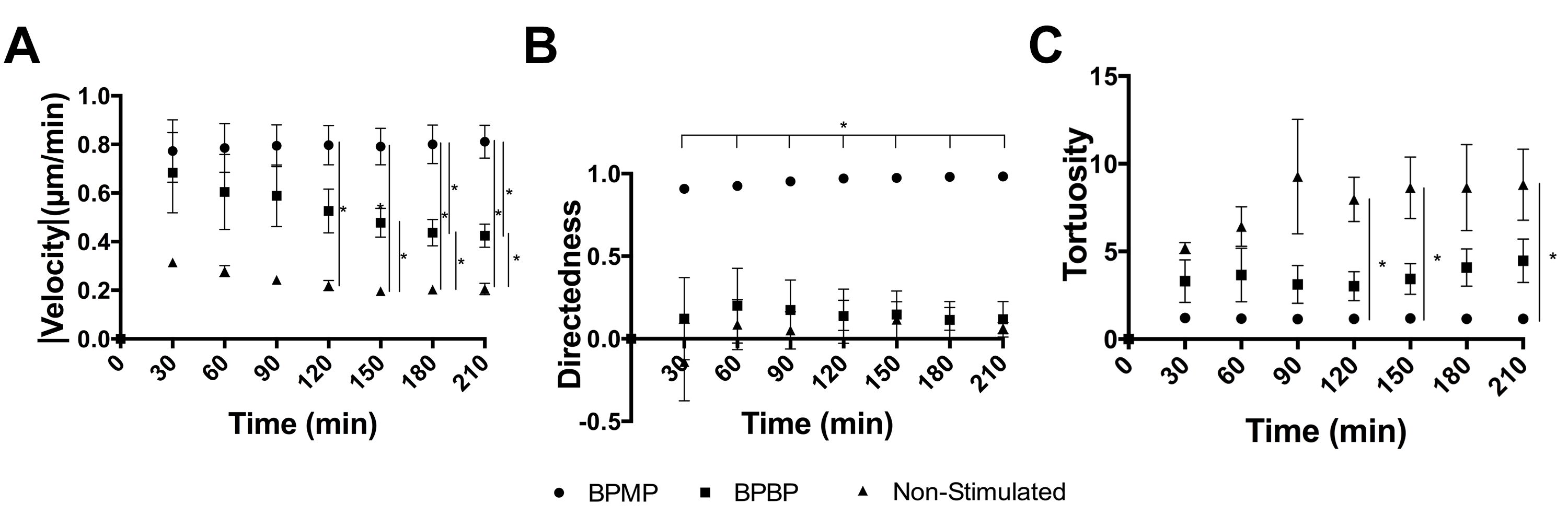

Supplement: Additional file 5: — is a figure showing that undifferentiated NPCs undergo rapid and cathode-directed galvanotaxis in the presence of BPMP stimulation. (A, B, C) The |velocity| (A), directedness (B) and tortuosity (C) of migration of undifferentiated NPCs are plotted in 30-minute intervals over the course of time-lapse imaging. The average |velocity|, directedness, and tortuosity at each time point tx, where x is in increments of 30 minutes, were calculated based on t = 0 as the initial time point. Tracking was performed for up to 210 minutes – the maximum amount of time that was common between all undifferentiated NPC groups. n = 3 for each group, *P <0.05. [file 13287_2015_49_MOESM5_ESM.tiff]

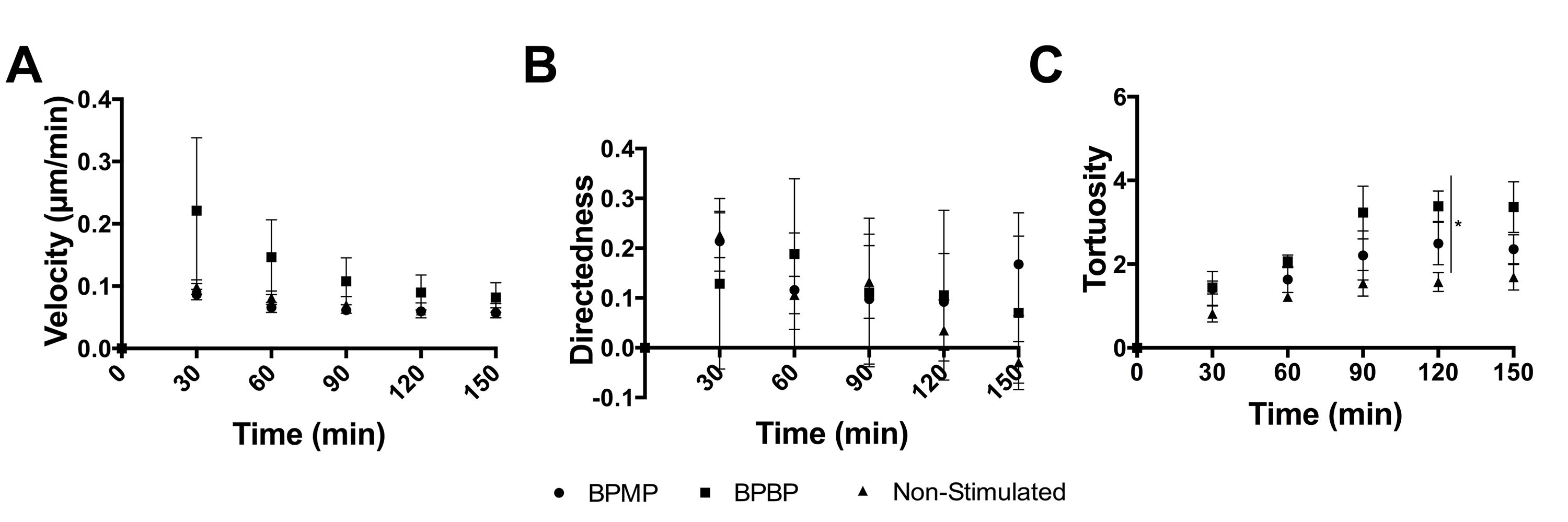

Supplement: Additional file 7: — is a figure showing that differentiated cells undergo negligible migration in the presence and absence of balanced biphasic stimulation. (A, B, C) The |velocity| (A), directedness (B), and tortuosity (C) of migration of differentiated cells are plotted in 30-minute intervals over the course of time-lapse imaging The average |velocity|, directedness, and tortuosity at each time point tx, where x is in increments of 30 minutes, were calculated based on t = 0 as the initial time point. Tracking was performed for up to 150 minutes – the maximum amount of time that was common between all differentiated cell groups. n = 3 for each group, *P <0.05. [file 13287_2015_49_MOESM7_ESM.tiff]
